# High levels of circulating folate concentrations are associated with DNA methylation of tumor suppressor and repair genes *p16*, *MLH1*, and *MGMT* in elderly Chileans

**DOI:** 10.1186/s13148-017-0374-y

**Published:** 2017-07-24

**Authors:** Hugo Sanchez, Mohammad B. Hossain, Lydia Lera, Sandra Hirsch, Cecilia Albala, Ricardo Uauy, Karin Broberg, Ana M. Ronco

**Affiliations:** 1 Unidad de Nutrición Pública, Instituto de Nutrición y Tecnología de los Alimentos Doctor. Fernando Monckeberg Barros (INTA), Universidad de Chile, El Líbano 5524, Macul, Santiago, Chile; 2 Division of Occupational and Environmental Medicine, Lund University, Lund, Sweden; 3 Institutet of Environmental Medicine, Karolinska Institutet, Stockholm, Sweden; 4 Unidad de Nutrición Humana, Instituto de Nutrición y Tecnología de los Alimentos Doctor Fernando Monckeberg Barros (INTA), Universidad de Chile, Santiago, Chile

**Keywords:** Folates, DNA methylation, Tumor suppressor genes

## Abstract

**Background:**

Changes in DNA methylation, one of the most studied epigenetic mechanisms, are considered an initial marker for early cancer detection. We evaluated how availability of dietary factors (folates and vitamin B12) involved in one-carbon metabolism may contribute to DNA methylation changes of cancer-related genes in human subjects.

**Methods:**

We studied, by pyrosequencing, the methylation of tumor suppressor gene *p16*, DNA repair genes *MLH1* and *MGMT*, and the repetitive element LINE-1 (as a surrogate for global DNA methylation), in blood of elderly individuals (*n* = 249) who had been exposed to folic acid (FA) through FA-fortified wheat flour during the last 12 years.

**Results:**

We found that serum folate and to a lesser extent, vitamin B12 concentrations, were significantly correlated with DNA methylation of *p16*, *MLH1*, and *MGMT*, but not with *LINE-1*. High serum folate concentrations (>45.3 nmol/L) were present in 31.1% of the participants. Although the methylated fraction of CpG sites in *p16*, *MLH1*, and *MGMT* was low (1.17–3.8%), high folate concentrations were significantly associated with methylation at the 3rd tertile of specific CpG sites in all genes with OR between 1.97 and 4.17.

**Conclusions:**

This study shows that a public policy, like food fortification with FA that increases circulating serum folate levels, could affect methylation levels of specific genes linked to cancer risk. Our present results deserve additional studies to clarify the real impact of high FA levels for risk of cancer in a whole population chronically exposed to a fortified food such as wheat flour.

**Trial registration:**

ISRCTN 48153354 and ISRCTN 02694183

## Background

Folates are hydrosoluble vitamins naturally present in foods. Folates are essential during pregnancy due to their role in prevention of neural tube defects and in many physiological functions, including cell proliferation and differentiation processes, DNA replication, angiogenesis, methylation reactions through the one-carbon metabolism, and antioxidant protection [1]. Due to the essentiality of folates during pregnancy, several countries have implemented fortification programs with folic acid (FA), the oxidized synthetic compound used in dietary supplements and food fortification. Chile started the fortification program in 2000 with 220 μg of FA/100 g of wheat flour, which led to a 40% decrease in the prevalence of neural tube diseases (NTD) 6 years later [2]. However, this public policy has increased folate consumption across the whole population, since bread is one of the most consumed foods in Chile [3]. In previous work, we have detected that 30% of the elderly people register circulating levels of folates higher than 48 nmol/L [4, 5], slightly higher than the upper physiological reference value of 45.3 nmol/L described for adults [6]. Some years ago, it was suggested that FA flour fortification masked vitamin B12 (Vit B12) deficiency found in elderly people [4, 5]; this evidence led to the health authorities to supplement elderly population with this vitamin [7]. Vit B12 is required for one-carbon metabolism and plays a key role in maintaining central and peripheral nervous system function [8]. Low Vit B12 status occurs more frequently in older adults, and its global deficit in this group ranges from 7 to 51% [5, 9]. Low Vit B12 concentration combined with high serum folates has been associated with increased risk of anemia, cognitive impairment, and neuroconduction alterations in older adults [10, 11].

Several biological functions of folates and Vit B12 are related with their participation in the one-carbon cycle that produces methyl groups for various processes in the body, such as DNA methylation, one of the key epigenetic processes in vertebrates [12]. Epigenetic regulation plays a critical role in normal growth and embryonic development by controlling the transcriptional activities of several genes [13]. A growing number of epigenetic changes have been reported in the regulation of key genes involved in cancer and aging [14]. DNA methylation changes seem to be an early event in tumorigenesis and have therefore been proposed as potential markers for early cancer detection [15]. Accumulating evidence indicates that DNA methylation markers in the stool and blood samples would provide a minimally invasive tool for colorectal cancer screening [16]. For this reason, an elevated intake of folates, mainly as FA, whose absorption in the gastrointestinal tract is not saturable, may lead to aberrant DNA methylation in cancer genes and may be not safe [17].

The Long Interspersed Nuclear Element 1 (LINE-1) retrotransposable elements make up about 17% of human DNA, and the methylation status of *LINE-1* is often used as a proxy for global DNA methylation [18]. The CpG sites in LINE-1 are usually heavily methylated and genome-wide loss of methylation from these sites has been regarded as a common epigenetic event in malignancy [19]. The *p16* tumor suppressor gene is found altered in a wide range of human cancers like endometrial carcinoma, hepatic, gastric, and colorectal cancer [20–22], and it has been suggested as a biomarker for colorectal cancer [23]. It encodes a specific inhibitor of cyclin-dependent kinase 4 and 6 and plays a pivotal role in tumor suppressor networks through inducing cellular senescence that acts as a barrier to cellular transformation by oncogenic signals [24]. Also, *p16* loses its expression mainly by the hypermethylation of its promoter [20, 21]. Methylated *p16* gene in blood may serve as a potential biomarker for cancer progression and a prognostic factor for several types of cancer [21].

*MLH1* promoter methylation has been implicated in the development of gastric and colorectal cancer; detection of MLH1 methylation in non-neoplastic gastric epithelia may be useful for screening patients who may be at risk of developing gastric cancer [25]. *MLH1* is an important component in the mismatch repair system [26], and a recent review highlighted the importance of epigenetic inactivation of *MLH1* gene in colorectal cancer [27]. Also, *MGMT* is crucial for genome stability. It repairs the naturally occurring mutagenic DNA lesion and prevents mismatch and errors during DNA replication and transcription. A meta-analysis involving 14 studies showed that the frequency of *MGMT* methylation was significantly higher in colorectal cancer than in normal tissues [28].

We have considered the chance that the increased incidence of colon cancer observed in Chile in the last years may be related with a high intake of FA due to food fortification with FA [29]. However, to date, there is no enough evidence to associate high FA consumption with cancer.

This report is a cross-sectional study aimed at evaluating epigenetic effects of high folate serum concentrations along with low, normal, or high Vit B12 serum concentrations on cancer-related genes and repetitive DNA sequences. The study was conducted in an elderly population in Chile who has been exposed to FA through fortified wheat flour during the last 12 years. We hypothesized that subjects with higher circulating concentrations of folates had increased levels of methylation in the tumor suppressor genes, to contribute with additional evidence related to the association between FA supplementation and colon cancer.

## Methods

### Subjects

We included elderly Chileans that were enrolled for a larger randomized controlled study in Santiago, Chile. The detail of the protocol was published previously [30]. The elderly were apparently healthy, free living, and beneficiaries of a complementary feeding program for older people above 65 years (called PACAM) [31]. Exclusion criteria were as follows: severe cognitive impairment (Mini-Mental State Examination score <19 points [32]); diabetes type 2 (fasting glucose concentrations ≥126 mg/dL, insulin, or use of other medications for diabetes), advanced renal impairment (creatinine clearance ≤30 mg/mL); hypothyroidism (TSH concentration ≥6 mIU/L); gastrointestinal surgery; alcohol abuse based on reported daily consumption; or a clinical history of stroke.

Participants of the study had been exposed to FA through fortification of wheat flour during the last 12 years since Chile started the nation-wide fortification program in the year 2000. Blood samples were collected and stored for blood measurements and DNA extraction. Ethics committees at the Institute of Nutrition and Food Technology (INTA) of the University of Chile and the Ministry of Health (Government of Chile) approved the study. All participants provided full written informed consent before being enrolled in the study and approved a consent to publish results.

### Analyses in blood

Serum concentrations of folates, Vit B12, methyl malonic acid (MMA), and holotranscobalamin (HoloTC) were measured. MMA and HoloTC were used as sensitive tests for early or mild vitamin B12 deficiency. Total homocysteine (tHcy) in plasma was used as an indirect indicator of folate, vitamin B12, and B6 deficiency. Serum Vit B12 and folate concentrations were assessed in duplicate with the use of the SimulTRAC_SNB radioassay kit (57Co/Folate125I; MP Diagnostics) (coefficient of variance (CV) 15–20%) [11]. Serum MMA was analyzed with the use of ultraperformance liquid-chromatography–tandem mass spectrometry [33](CV 5%). Serum HoloTC was determined with the Axis-Shield HoloTC ELISA (Axis-Shield Diagnostics Ltd.) (CV 10%), and plasma tHcy was determined by HPLC with a fluorescence detector (Agilent 1200; Agilent) (CV 10–15%).

All serum and plasma markers were measured at the Department of Agriculture, Agricultural Research Services, Western Human Nutrition Research Center, Davis, CA, USA.

### DNA isolation and methylation analysis

DNA was isolated from whole blood by DNeasy Blood & Tissue kit (Qiagen) and quantified by Qubit dsDNA BR kit (Life Technologies) by the NanoQuant Infinite M200Pro (Tecan). DNA integrity was checked by agarose gels stained by ethidium bromide. Four hundred nanograms of DNA (20 ng/μL) was bisulfite-treated using EZ DNA Methylation kit (Zymo Research, catalog number D5008). Bisulfite treatment converts unmethylated cytosine into uracil residues, leaving the methylated cytosine unchanged. The bisulfite-treated DNA was stored at −20 °C until used in PCR. We verified bisulfite conversion using non-CpG cytosine residues as built-in controls, and complete conversion of cytosine at a non-CpG site indicated successful bisulfite conversion.

**Fig. 1:**
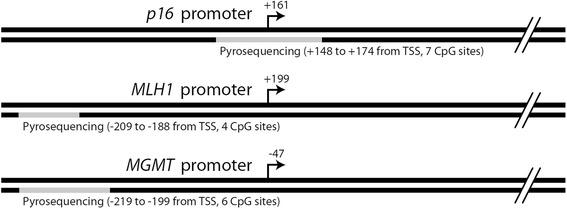
Pyrosequencing amplicons of *p16*, *MLH1*, and *MGMT* relative to known landmarks, including transcription start sites (TSS)

We studied, by pyrosequencing, the methylation of tumor suppressor genes (Fig. 1). PCR and pyrosequencing: bisulfite-treated DNA (0.6 μL) was used in a 15-μL PCR reaction using the Pyromark PCR kit (Qiagen). One of the PCR primers was biotinylated. The PCR product was purified using Streptavidin Sepharose High Performance beads (Amersham Biosciences, Uppsala, Sweden). The Sepharose beads containing immobilized PCR products were purified, washed, and denatured with 0.2 M NaOH and washed again using a vacuum prep tool (Pyrosequencing Inc., Westborough, MA, USA). Twelve microliters of the pyrosequencing primer (0.3 μmol/L) was annealed to the purified single-stranded PCR product, and pyrosequencing was done using the PSQ HS96 Pyrosequencing System (Qiagen).

Commercially available assays (Qiagen) were used to measure the methylation of *LINE-1* (four CpG sites between 305 and 331 bp, GenBank accession no. X58075), *p16* (seven CpG sites between +148 and +174 bp around the transcription start site in exon 1; L27211), and *MLH1* (4 CpG sites between −209 to −188 bp from transcription start site; U07418) following the manufacturer’s instruction [34, 35]. To determine the methylation level of *MGMT* promoter region, we developed an assay covering six CpG sites (−219 to −199 bp from transcription start site based on previous publications [36, 37], ENSG00000170430). The assay was designed by PyroMark Assay Design 2.0 software (Qiagen). The sequences of forward, reverse, and sequencing primers are GGTTATTTGGTAAATTAAGGTATAGAGTT, biotin- AACTATCCCAACATATCC, and GTTGTAGGAGGATTAGG, respectively.

We performed extensive quality control for the *p16* and *MLH1* assays as well as tested for amplification bias [38] and found that both the assays were within recommended range for this type of bias [35].

### Statistical analyses

Continuous variables were expressed as mean ± SD (standard deviation) and 95% confidence intervals (95% CI) or as median (p25–p75) and 95% CI. Categorical variables were expressed as percentages and 95% CI. The difference between sexes was calculated by two-sample mean-comparison test, two-sample Wilcoxon rank-sum test or Pearson’s Chi^2^ test, depending on the kind of variables. Spearman correlations were calculated for blood concentrations of vitamins and metabolites and the methylation in specific CpG sites.

We categorized vitamin B12 plasma levels as deficit (B12 < 148 pmol/L), marginal deficit (B12 ≥ 148 and <221 pmol/L), and normal (B12 ≥ 221 pmol/L) as suggested by Morris et al. [10]. Folates were categorized using the cut off points suggested by Pfeifer (as supra physiologic folate level ≥45.3 nmol/l) [6]. A logistic regression served to analyze the association between Vit B12 and folate and the percentage of methylation in specific CpG sites. CpG methylation was categorized in tertiles being the 3rd tertile the most methylated [26]. These results were adjusted by age, sex, body mass index (BMI), tobacco, and vitamin metabolites (HoloTC, tHcy, and MMA). We considered that tumor suppressor and DNA repair-related genes would be highly expressed thus conferring protection from risk of cancer. All statistical analyses were performed using STATA 14 software (StataCorp. 2015. Stata Statistical Software, Release 14. College Station, TX, StataCorp LP).

## Results

### Subjects

Descriptive data for the participants are shown in Table 1. The subjects were a representative sample of the respective age group within the population of low socio economic level elderly Chileans [9] with a relatively low education level (median 6 years); no sex differences were noted. Women consumed twice as much antidepressant and antihypertensive drugs than men (*p* < 0.001). Cognitive status assessed by the Mini Mental State Examination (MMSE) [32] was lower in women than in men; however, that difference was clinically irrelevant; men and women had scores within the accepted normal range for age (MMSE ≥21–22). BMI was slightly higher in women than in men (*p* < 0.05). Smoking rates was low in the participants and however higher in men compared to women (*p* < 0.05).

**Table 1:** Characteristics of subjects

	Men (*n* = 77)	Women (*n* = 172)	Total (*n* = 249)
Age (years)^a^			
mean ± SD	73.5 ± 2.7	73.9 ± 3.1	73.8 ± 3.0
95% CI	72.9–74.1	73.5–74.4	73.4–74.2
Education (years)			
median (p25–p75)^b^	6 (4–8)	6 (8–3)	6 (8–3)
≤6 years % (*n*)^c^	56.6 (43)	63.7 (107)	61.5 (150)
95% CI	44.7–67.9	55.9–71.0	55.1–67.6
Medication (number)***^c^			
0, % (*n*)	23.4 (18)	11.1 (19)	14.9 (37)
95% CI	14.5–34.4	6.8–16.7	10.7–19.9
1, % (*n*)	19.5 (15)	10.5 (18)	13.3 (33)
95% CI	11.3–30.1	6.3–16.0	9.3–18.1
2–3, % (*n*)	36.4 (28)	34.9 (60)	35.3 (88)
95% CI	25.7–48.1	27.8–42.5	29.4–41.6
4+, % (*n*)	20.8 (16)	43.6 (75)	36.6 (91)
95% CI	12.4–31.5	36.1–51.4	30.5–42.9
MMSE*^b^			
median (p25–p75)	28 (26–29)	27 (25–29)	27 (25–29)
BMI*^a^			
BMI*^a^ mean ± SD	26.9 ± 3.2	28.5 ± 5.1	28.1 ± 4.7
95% CI	26.2–27. 7	27.7–29.3	27.4–28.6
Tobacco consumption**^c^			
% (*n*)	10.5 (8)	5.8 (10)	7.3 (18)
CI 95%	4.6–19.4	2.8–10.4	4.3–11.2

Medications frequently used in elderly: antihypertensive, anti-inflammatory, antidepressant

*BMI* Body Mass Index, *MMSE* Minimental state examination

*p < 0.05; **p < 0.01; ***p < 0.001

^a^Two-sample mean-comparison test

^b^Two-sample Wilcoxon rank-sum test

^c^Pearson’s Chi^2^ test

### Methylation of genes

Table 2 describes DNA methylation for all genes studied as the percentage of methylation. We observed high methylation of *LINE-1* sequences, as expected [39]. In general, the percentage of methylation of the tumor suppressor and DNA repair genes studied were low (1.1 to 4.17%), suggesting that these genes were highly expressed [40]. No differences related with sex were observed in methylation status.

**Table 2:** DNA methylation in specific CpG sites

Genes	CpG site	Men (*n* = 77)	Women (*n* = 172)	Total (*n* = 249)
*LINE-1*	1	86.2 (84.5–86.9)	85.75 (84.5–86.9)	85.8 (84.8–86.7)
	2	82.6 (82.3–82.8)	82.5 (82.2–82.8)	82.5 (82.3–82.7)
	3	80.875 (80.4–81.75)	80.9 (80.3–81.3)	80.9 (80.5–81.2)
	4	82.2 (81.3–83.2)	82.0 (81.6–82.4)	82.0 (81.7–82.4)
	Average	83.3 (82.8–83.8)	82.8 (82.6–83.3)	82.9 (82.65–83.4)
*p16*	1	3.36 (3.0–3.89)	3.46 (3.25–3.64)	3.44 (3.25–3.6)
	2	1.575 (1.16–1.9)	1.58 (1.45–1.87)	1.58 (1.46–1.79)
	3	1.88 (1.37–2.12)	1.84 (1.62–2.07)	1.85 (1.66–2.04)
	4*	2.2 (1.91–2.25)	1.88 (1.75–2.05)	1.96 (1.82–2.11)
	5	0.91 (0–1.60)	1.65 (1.41–1.92)	1.49 (1.34–1.77)
	6	0	0	0
	7	1.89 (1.38–2.23)	1.94 (1.73–2.12)	1.91 (1.71–2.09)
	Average	1.89 (1.66–2.06)	1.92 (1.78–2.02)	1.907 (1.79–2.01)
*MLH1*	1	0 (0–0.99)	0	0
	2	1.39 (0–2.35)	1.82 (0–2.10)	1.725 (0–2.07)
	3	0	0	0
	4^γ^	2.3 (1.75–2.80)	2.495 (1.96–2.84)	2.435 (1.97–2.77)
	Average	1.25 (0.70–1.38)	1.12 (0.74–1.31)	1.165 (0.81–1.31)
*MGMT*	1	3.345 (2.53–4.21)	4.01 (3.51–4.66)	3.8 (3.41–4.26)
	2	0 (0–1.65)	0 (0–1.88)	0
	3	0	0	0
	4	1.865 (1.38–2.31)	1.87 (1.61–2.26)	1.87 (1.61–2.15)
	5	0	0	0
	6	0	0	0
	Average	1.555 (1.10–2.11)	1.72 (1.39–1.87)	1.66 (1.44–1.87)

Results are expressed as percentages of methylation and 95% CI. Two-sample Wilcoxon rank-sum test *p = 0.04; ^γ^ *p* = 0.07; for other results *p* > 0.1

### Blood determinations

The concentrations of vitamins in serum are shown in Table 3. Deficient concentrations of Vit B12 (<148 pmol/L) were present in 11.1% of the participants while 78.4% showed Vit B12 concentrations ≥221 pmol/L, with 29% of them having high Vit B12 concentrations (>680 pmol/L). It is likely that these subjects were taking vitamin supplements. HoloTC was higher in women than in men (*p* = 0.029), although HoloTC concentrations in both men and women were within the normal range (40–200 pmol/L) [41]. MMA concentrations were within the normal range indicate Vit B12 deficiency (MMA ≤400 nmol/L) [42]. Serum folate concentrations were 36 nmol/L (median; p25–p75 = 24–253.8) slightly higher in women compared with men (*p* = 0.0499). Interestingly, 31.1% of the participants showed high serum folate concentrations (>45.3 nmol/L): 34.1% of women compared with 24.3% of men (*p* = 0.031). Homocysteine levels were within normal reference range (<15 μmol/L) [43] without sex differences.

**Table 3:** Blood level of vitamins and metabolites

	Men (*n* = 66)	Women (*n* = 142)	Total (*n* = 208)
Vitamin B12 (pmol/L) Median (p25–p75)	370.7 (221.9–683.0)	438.5 (215.4–767.5)	409.3 (218.8–729.3)
<148 pmol/L % (*n*) (95% CI)	10.6(7) (4.4–20.6)	11.3(16) (6.6–17.7)	11.1(23) (7.1–16.1)
148–221 pmol/L % (*n*) (95% CI)	12.1(8) (5.4–22.5)	9.9(14) (5.5–16)	10.6(22) (6.7–15.6)
>221 pmol/L % (*n*) (95% CI)	77.3(51) (65.3–86.7)	78.9(112) (71.2–85.3)	78.4(163) (72.1–83.8)
MMA (nmol/L) Median (p25–p75)	187 (145–247)	177.5 (143–269)	180 (143–266)
HoloTC (pmol/L) Median (p25–p75)	67.7 (46.8–121.3)	102.9** (56–171.3)	180 (143–266)
Folates (nmol/L) Median (p25–p75)	34.6 (20.3–44.9)	36.6* (25.3–61.5)	36 (24.253.8)
>45.3 nmol/L % (*n*) (95% CI)	24.3(18) (15.1–35.7)	34.1(57)^γ^ (27.0–41.9)	31.1(75) (25.3–37.4)
Homocysteine (μmol/L) Median (p25–p75)	11.3 (9.3–12.7)	10.6 (8.7–13.2)	10.8 (8.7–13.1)

Results are expressed as median (p25–p75) and % (*n*) and 95% CI

*MMA* methylmalonic acid, *HoloTC* holotranscobalamin

*p = 0.0499, two-sample Wilcoxon rank sum test; **p = 0.029; ^γ^ *p* = 0.031, Chi^2^ test

### Analysis of correlations and regression models

Table 4 shows correlations between the vitamins and DNA methylation in studied genes. No correlation was found between vitamins and methylation in *LINE-1*. Vit B12 and folates correlated with DNA methylation in specific CpG sites of *p16*, *MGMT*, and *MLH1* genes. For *p16*, Vit B12 was positively correlated with DNA methylation in CpG 1, 3, and 5 and with average methylation, and folates correlated positively with CpG sites 3 and 5. For *MLH1*, Vit B12 correlated negatively with methylation of CpG site 1, and folates correlated positively with CpG 2, 4 and average *MLH1* methylation. For *MGMT*, Vit B12 correlated positively with CpG 2, and folates correlated positively with methylation of CpG sites 1–3 and average and negatively with CpG4. Concentrations of HoloTC, Hcy, and MMA did not correlate with methylation in the DNA of any studied gene.

**Table 4:** Spearman correlation for blood concentrations of vitamins and metabolites and DNA methylation in specific CpG sites of *LINE-1*, *p16*, *MLH1*, and *MGMT *genes

	CpG site	Vit B12 *n* = 242	Folates *n* = 241	HoloTC *n* = 193	Hcy *n* = 193	MMA *n* = 193
*LINE-1*	1	−0.1064	0.0193	−0.1357	0.0509	0.0298
	2	−0.0106	−0.1259	−0.0680	−0.0811	0.1004
	3	0.1067	0.0360	0.1321	−0.0644	−0.0318
	4	−0.0346	−0.0127	−0.1313	0.0281	0.0394
	Average	−0.0373	0.0302	−0.1135	0.0604	0.0166
*p16*	1	0.1888*	0.0156	0.1152	−0.0002	−0.0137
	2	−0.0552	−0.1519	0.0104	−0.0239	−0.0303
	3	0.2965*	0.2320*	0.1137	−0.1293	−0.1288
	5	0.2198*	0.2974*	0.0595	−0.0462	−0.1619
	6	−0.1668	−0.1787	−0.0167	0.0709	0.0449
	7	0.0863	−0.1029	0.1230	0.0455	−0.0597
	Average	0.2288*	0.0867	0.1553	−0.0880	−0.1318
*MLH1*	1	−0.2809*	−0.1616	0.0360	−0.0555	−0.0634
	2	0.0977	0.2453*	−0.0248	−0.0422	−0.0656
	3	−0.1404	−0.1102	−0.0374	−0.0393	−0.0451
	4	0.1246	0.3121*	0.0399	−0.1387	−0.1486
	Average	−0.1081	0.1786*	0.0188	−0.1410	−0.1478
*MGMT*	1	0.1118	0.2291*	−0.0167	−0.0589	−0.0211
	2	0.3142*	0.4196*	−0.0082	−0.0702	−0.1504
	3	0.0791	0.2007*	−0.0522	−0.0417	−0.0876
	4	−0.1561	−0.3457*	0.0002	0.0808	0.1207
	5	−0.1187	−0.1011	−0.0570	−0.0621	−0.0806
	6	−0.0111	−0.0272	−0.0012	−0.1773	−0.1511
	Average	0.1168	0.2489*	−0.0591	−0.0567	−0.0474

*p < 0.01

A logistic regression model (Table 5) was used to evaluate possible associations between high folate (>45.3 nmol/L) and low Vit B12 concentrations (< 148 and between 148 < 221 pmol/L) with DNA methylation of tumor suppressor and repair genes above the 3rd tertile that might lead to a lower expression. For *p16*, folate concentrations >45.3 nmol/L were significantly associated with risk of increased DNA methylation of CpG sites 3 and 5, with an OR of 1.97 and 4.17, respectively (Table 5). For *MLH1*, high folate level was significantly associated with risk of increased mean DNA methylation (OR: 2.2) and also to increased DNA methylation of CpG sites 1–3 (OR: 2.31, 3.73, and 2.08, respectively) (Table 5). For *MGMT*, high folate level was associated risk of increased average DNA methylation (OR: 2.84) and increased DNA methylation of CpG site 4 (OR: 3.91). No significant associations were observed between low Vit B12 (<148 nmol/L) and DNA methylation.

**Table 5:** Logistic Regression Model between vitamin B12 and folate levels and the risk of methylation of *p16*, *MLH1*, and *MGMT* at the 3rd tertile (%)

				Vit B12 (pmol/L)		Folates* (nmol/L)
				<148	148–221	>45.3
*p16*	CpG site	3^a^	OR	0.70	0.36	1.97
			95% CI	0.23–2.08	0.10–1.23	1.04–3.75
		5^b^	OR	0.46	0.67	4.17
			95% CI	0.13–1.6	0.2–2.2	2.12–8.19
*MLH1*		1^c^	OR	0.56	0.93	2.31
			95% CI	0.14–2.19	0.28–3.13	1.15–4.64
		2^d^	OR	0.43	0.86	3.73
			95% CI	0.10–1.88	0.26–2.90	1.83–7.59
		3^e^	OR	1.18	1.22	2.08
			95% CI	0.34–4.13	0.38–3.95	1.03–4.20
		Average^f^	OR	0.99	1.60	2.20
			95% CI	0.26–3.80	0.48–5.32	1.08–4.50
*MGMT*		4^g^	OR	0.96	0.12	3.91
			95% CI	0.28–3.26	0.014–0.99	1.88–8.11
		Average^h^	OR	0.92	0.6	2.84
			95% CI	0.28–2.98	0.19–2.52	1.42–5.69

Adjusted by sex, age, tobacco, BMI, Hcy, HoloTC, and MMA

*OR* odd ratio, *CI* confidence interval

*p < 0.05 vs folate <45.3 nmol/L

^a^< 2.35% methylation

^b^2.11% methylation

^c^< 5.24% methylation

^d^< 2.65% methylation

^e^ < 0.34% methylation

^f^< 2.14% methylation

^g^< 3.2% methylation

^h^< 1.48% methylation

## Discussion

In this study, we found that about one third of the participants, who have been exposed to FA during 12 years through wheat flour fortification, show a mean circulating folate concentrations higher than 45.3 nmol/L, described as supra physiological [6]. More women than men registered supra physiological folate concentrations (*p* < 0.031). Also, circulating folate concentrations were slightly higher in women than in men (*p* < 0.0499). These results demonstrate that there is probably no folate deficiency in the Chilean population, including the elderly, which may be a consequence of the mandatory public policy of fortifying wheat flour with FA to prevent neonates NTD initiated in Chile the year 2000 [2]. Other important finding is that high serum folate levels increase DNA methylation of tumor suppressor genes *p16* and DNA repair genes *MLH1* and *MGMT* in blood. Folate concentrations >45.3 nmol/L increased the risk of methylation in the 3rd tertile in specific CpG sites of the genes *p16* (CpG 5, OR = 4.17), *MLH1* (CpG 2, OR = 3.73), and *MGMT* (CpG4, OR = 3.91). Also, high folate concentrations increased the risk of methylation in the average DNA methylation of *MGMT* and *MLH1*.

Due to the essentiality of folates during pregnancy, and to prevent NTD in newborns, several countries, including Chile, started food FA fortification programs to cover requirements of the target population (600 μg/day). However, this policy has led to elevate blood levels of the whole population; estimations are that 20 to 30% of the general population present serum folate concentrations over 40 nmol/L [29]. Previous studies found a high incidence of Vit B12 deficiency in elderly Chilean, and it was suggested that those deficiency was masked by high folate levels as a consequence of the food fortification policy of with folates [4, 5]. This evidence led to the Health Ministry of the Chilean government to implement a program for older adults, users of the public health system, which provided them periodically with milk or a packaged soup fortified with Vit B12. This may explain that in the present study, only 11.1% of the participants presented Vit B12 deficiency (Table 3) [7].

Increased circulating folate concentrations have also been considered a risk factor for cardiovascular disease [44] and some forms of cancer [45]. Epidemiologic studies carried out in the USA, Canada [46], and Chile [29] have reported a temporal relationship between mandatory fortification with FA and a greater incidence of colorectal cancer related to the beginning of the fortification programs.

Men with high blood folate levels were at greater risk of high-grade (more aggressive) prostate cancer compared with men with lower folate levels [47]. The Women’s Health Initiative Observational Study cohort provided new evidence that increased folate intake during the early post-fortification period may have been associated with a transient increase in colorectal cancer risk [48]. In a population-based study from northern Sweden, a bell-shaped relationship between plasma folate concentrations and colorectal cancer risk was observed, with a doubling of risk for subjects in the middle versus lowest quintile [49].

On the contrary, a recent study with more than 1400 older adults (≥57 years) with a median follow-up of 6.3 years showed that high total folate intake appear to be protective against cancer in post-FA fortification years. Those authors did not find significant associations between the presence of non-metabolized FA, intake of naturally-occurring food folate or FA separately, and cancer incidence [50].

Probably, folates like other compounds and vitamins may have a U shape effects, with hazardous effects with both low and high levels.

Folate homeostasis disruption (by high or low levels of folate intake) may change the one-carbon metabolism, the process responsible for production of methyl groups, leading to gene transcription alterations (overexpression and/or gene silencing) through DNA gene hyper- or hypo-methylation reactions. Disruption of epigenetic processes can in turn lead to altered gene and cell functions leading to malignancy. Recent advancements in the rapidly evolving field of cancer epigenetics have shown extensive reprogramming of every component of the epigenetic machinery in cancer, including DNA methylation [15].

It should be noted that we here analyzed associations between FA and DNA methylation in blood and we do not know if the observed changes in DNA methylation actually are present in the target tissues or associated with cancer incidence. Tumor suppressor genes become inactive with hypermethylation of their promoter regions, and aberrant promoter methylation of tumor suppressor genes is associated with different types of cancers [51] and its serum detection may be a good marker for cancer diagnosis [52]. Recent publications have found that in cervical, colon, breast, bladder, gastric, lung, and esophageal tumors, tumor suppressor genes like *p15, p16, p21, p27, p57*, and others are hypermethylated in their promoters [53]. Moreover, *p16* hypermethylation was found to be an independent prognostic marker for cancer-specific survival. Still, our hypotheses about the effects of folic acid and vitamin B12 on epigenetic processes in relation to tumor suppressor inactivation warrant further investigation and confirmation in future research following up the cancer incidence among individuals with varying tumor suppressor methylation in blood.

The availability of dietary factors involved in one-carbon metabolism may contribute to aberrant DNA methylation [54]. A recent study on 87 elderly (65–75 years) showed that long-term supplementation of FA and Vit B12 (2 years) resulted in effects on DNA methylation of several genes implicated in carcinogenesis and early embryonic development [55]; genome-wide methylation analysis revealed pronounced methylation changes in the DIRAS3, ARMC8, and NODAL genes. Further, among positions that were differentially methylated after the intervention with FA and Vit B12, CpG positions within CpG islands and around transcription start sites were overrepresented. This suggests that the availability of folate affects DNA methylation at specific DNA regions. Also, other factors that seem to affect DNA methylation, e.g., for p16 including tobacco, radiation exposure, and pollution, have been associated with aberrant methylation of this gene, and its detection in blood or in serum has been suggested to be a good marker for cancer diagnosis [25]. The percentages of DNA methylation in LINE-1, *p16*, and *MLH1* in the present study are comparable to the methylation levels found in younger Argentinean women exposed to arsenic, although they were on average slightly lower (Argentinean 3.4 vs. Chilean 1.9% for *p16*; Argentinean 4.2 vs. Chilean 1.17 [34, 35]. Significant correlation between folates and methylation at specific CpG sites of *p16* and *MLH1* were different from those linked to arsenic exposure; in this study, we found correlations with methylation of *p16* sites 3 and 5 and *MLH1* site 4, compared with sites 1 both in *p16* and *MLH1* associated with arsenic exposure. Stronger correlations were observed for methylation of several sites in *MGMT* with folates. This suggests that exposure to different compounds may disrupt DNA methylation at different genes and sites.

One limitation of this study is the lack of RNA to assess gene expression and the respective association with the level of DNA methylation, and that we did not follow the cancer incidence in this cohort. However, there is enough information to expect that a hypermethylated gene is less expressed than a hypomethylated gene [56]. A highlight of this study is the homogeneity of the sample, since all of the participants have been exposed to wheat flour fortification with FA.

In summary, we conclude that long time exposure to highly consumed FA-fortified foods by a non-target population increase blood concentrations of circulating folates inducing to a higher risk of higher gene-specific methylation levels. Although marginal, the increased methylation levels of tumor suppressor and DNA repair genes may reduce their gene expression that are in turn associated with increased risk of some cancers.

## Conclusions

Elderly Chilean exposed to fortified foods with FA during the last 12 years do not present folate deficiency and have normal or elevated levels of circulating folates. Folate concentrations >45.3 nmol/L increased the risk of methylation in specific CpG sites of tumor suppressor and DNA repair genes.

## Abbreviations

AF: Folic acid
CV: Coefficient of variance
HoloTC: Holotranscobalamin
LINE-1: Long interspersed nuclear element 1
MMA: Methyl malonic acid
MMSE: Mini-Mental State Examination score
NTD: Neural tube disease
OR: Odd ratio
tHcy: Total homocysteine
Vit B12: Vitamin B12
